# Association of oxidative stress biomarkers with primary congenital glaucoma

**DOI:** 10.1371/journal.pone.0328663

**Published:** 2025-08-13

**Authors:** Mingxi Shao, Yani Wan, Yingzhu Li, Yi Ma, Jun Ren, Shengjie Li, Wenjun Cao

**Affiliations:** Department of Clinical Laboratory, Eye & ENT Hospital, Shanghai Medical College, Fudan University, Shanghai, China; Georgetown University Medical Centre, UNITED STATES OF AMERICA

## Abstract

**Purpose:**

To explore the relationship between oxidative stress biomarkers and primary congenital glaucoma (PCG).

**Methods:**

This case-control study included 40 PCG patients and 38 matched controls. Serum total antioxidant status (TAS), as well as superoxide dismutase (SOD), malondialdehyde (MDA), reactive oxygen species (ROS) and hydrogen peroxide (H_2_O_2_) levels were measured, along with eye and body exams. Logistic regression analysis was performed for PCG risk factors and machine learning model biomarker diagnosis.

**Results:**

In the PCG group, H_2_O_2_ and MDA levels were notably higher than in controls (p < 0.001, **p* *= 0.020), while TAS levels were significantly lower (p = 0.043). Adjusting for age and gender, the serum TAS (OR = 0.07, 95% CI 0.01–0.85, *p* = 0.037), H_2_O_2_ (OR = 1.21, 95% CI 1.09–1.35, *p* = 0.001) and MDA (OR = 1.17, 95% CI 1.00–1.34, *p* = 0.034) were determined to be independent risk/protective factors for PCG. Pearson analysis revealed significant negative correlations: SOD with anterior chamber depth (r = −0.445, p = 0.012) and H_2_O_2_ with mean deviation values for the visual field (r = −0.412, p = 0.041). Positive correlations were also significant: MDA with axial length (AL (r = 0.576, p = 0.002). The XGBoost or KNN model using TAS alone achieved the highest AUC (0.74) in five-fold cross-validation.

**Conclusion:**

The decrease in TAS levels and the increase in H_2_O_2_ and MDA levels are found to be correlated with PCG, and the results indicate that oxidative stress plays a part in congenital glaucoma onset.

## 1. Introduction

Primary congenital glaucoma (PCG) is a frequent cause of blindness in the paediatric population, seriously affecting visual acuity and visual development in affected children [1]. The prevalence of PCG in Chinese children is approximately 0.0020% to 0.0035% [2], accounting for about 2% to 15% of cases of infant blindness worldwide [3]. The ultimate consequence of all types of glaucoma is damage to the optic nerve.

PCG comprises a series of eye diseases, all cause by the obstruction of trabecular network dysplasia, which then leads to a series of such clinical symptoms as increased intraocular pressure, corneal enlargement and optic nerve atrophy. However, the mechanisms behind the disease’s development remain unclear. Currently, most studies suggest that the main factors causing optic nerve damage in PCG include the mechanical injury hypothesis, the vascular hypothesis, the immune mechanism, genetic susceptibility and the body’s stress response [4,5]. Meanwhile, oxidative stress is caused by the accumulation of reactive oxygen species (ROS) and reactive nitrogen species (RNS) and by the imbalance between the body’s antioxidant defence and its detoxification capacity, which plays an important role in the development of PCG [6–9]. Recent studies highlight the contributions of oxidative stress to retinal ganglion cell damage in primary angle-closure glaucoma (PACG). For instance, Goyal et al. [10] note elevated superoxide dismutase (SOD) levels with a glutathione peroxidase concentration in PACG patients’ aqueous humor, contrasting results found among cataract patients. Chang et al. [11] also identified higher malondialdehyde (MDA) concentrations in PACG patients.

Despite recent findings, the association with oxidative stress biomarkers in congenital glaucoma has not been extensively studied. This study seeks to address this by examining the correlation between these biomarkers and PCG in a case-control format.

## 2. Methods

### 2.1 Patients

Conducted at the Department of Ophthalmology and Visual Sciences, Fudan University Eye and Ear, Nose and Throat (ENT) Hospital in Shanghai, China, this study was approved by the hospital’s Ethics Committee and followed the Declaration of Helsinki’s ethical guidelines. All participants participated in the study voluntarily after being fully informed of the purpose of the study. All participants signed their written informed consent.

From 1^st^ January 2022–1^st^ December 2023, PCG subjects were recruited from patients with a clear ophthalmic diagnosis among ophthalmic patients of the Eye and ENT Hospital of Fudan University. Controls were recruited from among annual health screening attendees, ensuring age and sex matching. The study aimed to compare oxidative stress-related factor levels between PCG patients and normal subjects and to explore potential new risk factors associated with oxidative stress in PCG. We use PASS to estimate the sample size. We set power = 0.8,α = 0.05, R1(Ratio |H1 = P1/P2), resulting in a sample size equal to 36. We included 38 cases and 40 controls. The data were accessed for research purposes on 17^th^ May 2024. Authors had access to information that could identify individual participants during or after data collection.

### 2.2 Inclusion and selection criteria

PCG is diagnosed by an ophthalmic glaucoma specialist according to clinical diagnostic criteria [12]. Thus, the inclusion criteria for congenital glaucoma usually cover the following [13]:(1) clinical signs of PCG evident at 0–3 years of age; (2) eye pressure test over 21 mmHg (1 mmHg = 0.133 kPa), enlarged or depressed visual cup, narrowed disk edge and asymmetrical or progressively increasing cup-to-disc ratio; (3) broadening of the cornea’s circumference or corneal hydration; (4) progressive myopia, or the rate of eye enlargement exceeds the normal growth rate, corresponding to glaucomatous optic neuropathy, with visual field defects that can be repeatedly detected and other lesions causing visual field defects excluded; while also ruling out other diseases that may cause similar symptoms, such as keratopathy, trauma, etc.

A total of 45 control subjects were recruited, of whom 7 normal subjects were later excluded from the study based on the inclusion criteria. The final sample consisted of 38 control subjects who met the following criteria: (1) no history of any type of glaucoma or any other eye disease; (2) no history of systemic diseases, such as acute infectious diseases, kidney disease, autoimmune disease, cancer.

According to the diagnosis and inclusion and exclusion criteria, eight PCG patients were removed (two cases of PACG, three cases of other eye diseases and three cases of eye surgery), and seven normal control patients were removed (four cases of eye disease and three cases of other systemic diseases). Finally, 40 PCG patients and 38 normal controls in total were included.

### 2.3 Examination

The study systematically collected clinical data from patients diagnosed with PCG, who then underwent a comprehensive eye exam by a glaucoma specialist. The examination included Goldmann tonometry, slit-lamp microscopy, B-ultrasound, central perimetry (Humphrey or Octopus perimetry), peripheral perimetry (Goldmann perimetry) and axial length measurement (A-ultrasound). Normal control subjects also received a preliminary eye exam to compare ocular characteristics with PCG patients, ensuring the study’s accuracy and reliability.

### 2.4 Laboratory detection

All laboratory analyses were performed in the Clinical Laboratory Department of the Eye and ENT Hospital of Fudan University. Subjects provided blood specimen by venipuncture in the anterior cubital fossa in the early morning. Thereafter, serum derived from the blood samples was processed within the first hour after collection and then stored at a temperature of −80°C for future use. SOD levels were determined using the ultraviolet (UV) enzymatic method and the total antioxidant status (TAS) was assessed via colorimetry, both using commercial kits provided by JiuQiang biotechnology from Beijing, China. These assays were executed on the Roche Diagnostics Cobas 8000 system, which is based in Mannheim, Germany.

In adherence to the instructions for the MDA detection kit (S0131S) provided by China Nantong Biyuntian Biotechnology Company, MDA detection was performed on serum samples, and the MDA concentration was measured using a multi-mode microplate reader, specifically for the US Biotek SynergyH1 model, with the detection wavelength set at 532 nm. In addition, the same serum samples were analysed for hydrogen peroxide (H_2_O_2_) content using the hydrogen peroxide detection kit provided by Beyotime (09019T). H_2_O_2_ levels in each serum sample were measured at 560 nm using the same multimode microplate reader.

### 2.5 Machine learning development

In this study, logistic regression(LR), random forest model(RF), K-Nearest Neighbor (KNN), support vector machine (SVM) model, Adaboost model and XGBoost model were used to predict serum TAS, SOD, MDA and TAS + SOD + MDA levels in patients with PCG, respectively. To optimise the prediction model, a combination of grid search and manual fine-tuning was used to obtain the final hyperparameters, and the machine learning model underwent training and validation through a five-fold cross-validation process. The dataset is randomly split into five folds with similar data distribution. The model is trained on four folds and validated on the remaining one. This process is repeated five times, using each fold as the validation set once. Finally, the performance of the five machine learning models was evaluated through a comprehensive comparison of metrics, including the areas under the curve (AUC), sensitivity, specificity, positive and negative predictive values, accuracy and F1 score.

### 2.6 Statistical analysis

The results are detailed as mean±standard deviation (SD) and statistically examined with SPSS version 19.0. Graphs were made using GraphPad Prism 6, and data normality was determined by applying the Kolmogorov–Smirnov test.

In the context of the case-control study, a range of statistical tests was appropriately applied to compare patient characteristics across the groups. The data analysis was conducted by employing an independent student t test and a chi-square (χ^2^) test. In addition, a binary logistic regression model was applied to estimate odds ratios (ORs) with 95% confidence intervals (CIs), adjusting for covariates, including age, sex, local glaucoma medication usage and intraocular pressure (IOP). In addition, Spearman’s correlation analysis assessed the relationship among IOP, vertical cup-to-disc ratio (VCDR) and oxidative stress biomarkers.

## 3. Results

### 3.1 Basic patient information

In total, 40 PCG patients (male = 22, female = 18) and 38 normal controls (male = 16, female = 22) were selected based on screening criteria. Table 1 provides a detailed outline of their demographic and ocular features.

**Table 1:** Demographic and ocular features.

Variable	Control group (n = 38)	PCG group (n = 40)	*p*-value
Age	18.92 ± 12.93	18.90 ± 13.98	0.995
Gender (male/female)	16/22	22/18	0.365
TAS, mmol/L	1.45 ± 0.19	1.35 ± 0.21	0.043
SOD,U/ml	157.79 ± 20.74	154.95 ± 22.76	0.567
H_2_O_2_, µM	322.46 ± 4.40	328.59 ± 7.67	<0.001
MDA, µM	10.97 ± 3.50	12.93 ± 3.65	0.020
IOP, mmHg	–	25.20 ± 11.59	
MD, dB	–	17.53 ± 10.24	
AL, mm	–	26.31 ± 2.25	
ACD, mm	–	3.29 ± 0.47	
CCT, mm	–	557.83 ± 71.10	
Duration	–	31.14 ± 34.83	
VCDR	–	0.82 ± 0.16	
MS, dB	–	12.60 ± 9.52	

Abbreviations: Independent-samples t-test; #Mann-Whitney U test;* χ² test. VCDR: vertical cup-disc ratio; CCT: central corneal thickness; ACD: anterior chamber depth; AL: axial length; MD: visual field meandeviation; IOP:intraocular pressure; MS: visual field mean sensitivity; SOD: superoxide dismutase; TAS: total antioxidant status; H_2_O_2_: hydrogen peroxide; MDA: malondialdehyde.

**Fig 1:**
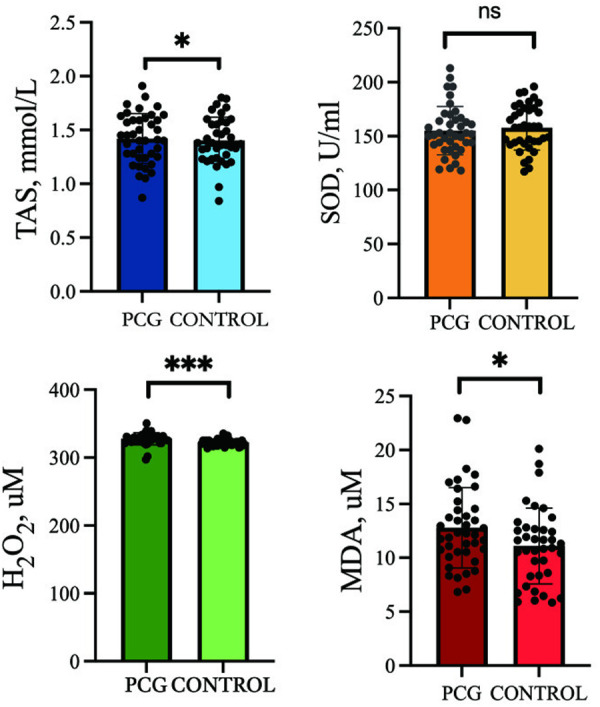
The level of Oxidative stress biomarker in PCG and control groups. Abbreviations: SOD: superoxide dismutase; TAS: total antioxidant status; H_2_O_2_: hydrogen peroxide; MDA: malondialdehyde; * p < 0.05; ** p < 0.001; ns p > 0.05.

The PCG and control groups did not significantly differ in average age, gender distribution and SOD levels (p > 0.050), whereas one characteristic of PCG subjects was identified to be a significant rise in H_2_O_2_ (p < 0.001) and MDA (p = 0.020) levels relative to the controls. The serum TAS level in the PCG group was significantly lower than that in the control group, indicating statistical significance (p = 0.043) (Table 1, Fig 1).

### 3.2 Comparison of oxidative stress biomarker in subjects with PCG according to severity

According to MD (MD < 12 and MD ≥ 12), the PCG subjects were categorized into 2 subgroups: mild+moderate (N = 8), severe (N = 17) (Table 2). However, the difference of SOD, TAS, H_2_O_2_ and MDA were not statistically significant between the two subgroups.

**Table 2:** Comparison of oxidative stress biomarker in subjects with PCG according to severity.

	MD < 12dB	MD ≥ 12dB	*p* Value
TAS(mmol/L)	1.34 ± 0.32	1.49 ± 0.24	0.157
SOD (U/ml)	157.88 ± 24.87	154.71 ± 17.36	0.451
H_2_O_2_ (uM)	331.92 ± 8.55	328.11 ± 5.46	0.557
MDA (uM)	13.36 ± 2.06	13.68 ± 4.56	0.094

Abbreviations: SOD: superoxide dismutase; TAS: total antioxidant status; H_2_O_2_: hydrogen peroxide; MDA: malondialdehyde; MD: visual field meandeviation.

### 3.3 Logistic regression analysis of oxidative stress biomarker correlations between PCG and control groups

A binary logistic regression analysis, adjusted for age and gender, identified serum levels of TAS (OR = 0.07, 95%CI = 0.01–0.85, *p* = 0.037), H_2_O_2_ (OR = 1.21, 95%CI = 1.09–1.35, *p* = 0.001), and MDA (OR = 1.17,95%CI = 1.01–1.34, *p* = 0.034) as independent risk/protective factors for PCG, such that PCG initiation are linked in the study to oxidative stress (Table 3).

**Table 3:** Logistic regression analyzed the link between oxidative biomarkers and PCG risk.

	Non-adjusted	Adjust II
TAS	0.09 (0.01, 0.97) 0.047	0.07 (0.01, 0.85) 0.037
SOD	0.99 (0.97, 1.01) 0.562	1.00 (0.97, 1.02) 0.642
H_2_O_2_	1.20 (1.08, 1.34) 0.001	1.21 (1.09, 1.35) 0.001
MDA	1.17 (1.02, 1.35) 0.026	1.17 (1.01, 1.34) 0.034

Adjust II adjust for: sex and age. Abbreviations: TAS: total antioxidant status; SOD: superoxide dismutase; H_2_O_2_: hydrogen peroxide; MDA: malondialdehyde.

### 3.4 Pearson correlation test between oxidative stress biomarkers and ophthalmic parameters

A Pearson analysis demonstrated significant negative correlations between SOD and ACD (r = 0.445, *p* = 0.012) and between H_2_O_2_ and MD (r = 0.412, *p* = 0.041). In addition, the MDA level was positively correlated with AL (r = 0.576, *p* = 0.002) (Table 4).

**Table 4:** The Pearson correlation analysis between oxidative stress biomarkers and ophthalmic parameter.

	IOP	MD	AL	ACD	CCT	Duration	VCDR	MS
TAS	R = −0.061 *p* = 0.733	R = 0.147 *p* = 0.503	R = −0.080 *p* = 0.712	R = 0.172 *p* = 0.372	R = 0.142 *p* = 0.530	R = 0.123 *p* = 0.490	R = 0.240 *p* = 0.238	R = −0.294 *p* = 0.268
SOD	R = −0.019 *p* = 0.913	R = 0.075 *p* = 0.720	R = −0.280 *p* = 0.168	R = −0.445 *p* = 0.012	R = −0.052 *p* = 0.810	R = −0.061 *p* = 0.725	R = −0.117 *p* = 0.553	R = −0.184 *p* = 0.480
H_2_O_2_	R = −0.070 *p* = 0.688	R = −0.412 *p* = 0.041	R = 0.173 *p* = 0.398	R = −0.213 *p* = 0.250	R = 0.218 *p* = 0.307	R = 0.103 *p* = 0.550	R = −0.061 *p* = 0.760	R = 0.273 *p* = 0.290
MDA	R = 0.323 *p* = 0.055	R = −0.186 *p* = 0.374	R = 0.576 *p* = 0.002	R = 0.032 *p* = 0.866	R = 0.171 *p* = 0.424	R = 0.211 *p* = 0.217	R = −0.123 *p* = 0.532	R = −0.031 *p* = 0.907

Abbreviations: IOP:intraocular pressure; VCDR: vertical cup-disc ratio; CCT: central corneal thickness; ACD: anterior chamber depth; AL: axial length; MD: visual field mean deviation; MS: visual field mean sensitivity; SOD: superoxide dismutase; TAS: total antioxidant status; H_2_O_2_: hydrogen peroxide; MDA: malondialdehyde.

### 3.5 Developing a machine learning model to diagnose PCG

**Fig 2:**
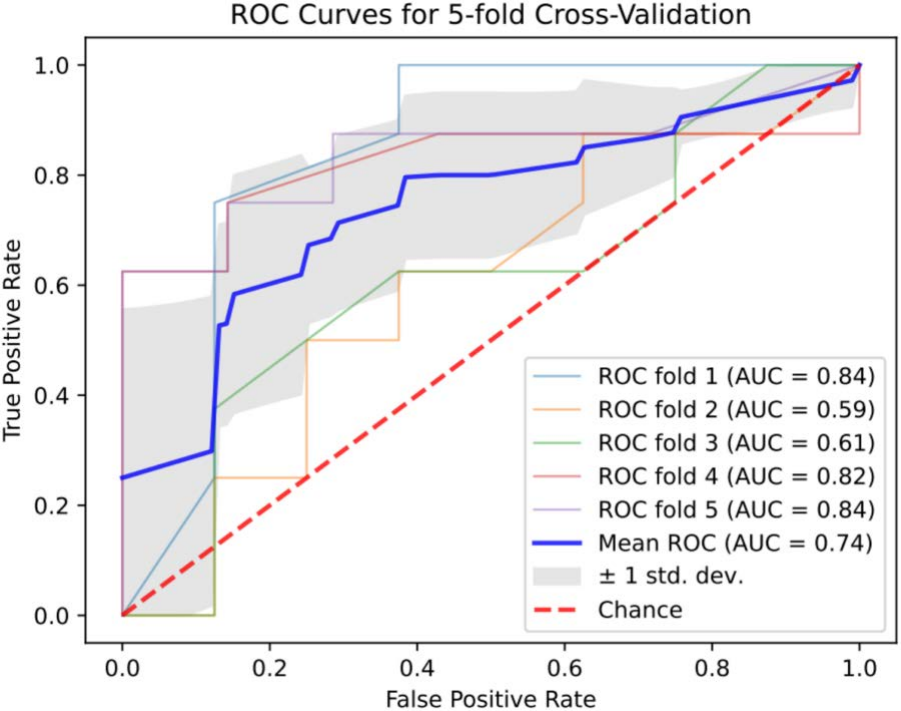
ROC curves of TAS + SOD + MDA to diagnose PCG.

The experimental and control groups’ four indicators—TAS, SOD, MDA and the combination of TAS + SOD + MDA—were incorporated into five machine learning models. The Table 4 presents six performance evaluation metrics for the logistic regression, random forest, KNN, SVM, Adaboost, and XGBoost models. It can be observed that the latter, constructed using the single indicator TAS, has the highest AUC value (0.74). Meanwhile, the TAS index demonstrated higher AUC values, sensitivity, specificity, positive and negative predictive values, accuracy and F1 scores compared to the combined SOD, MDA and TAS + SOD + MDA indices (Table 5; Fig 2).

**Table 5:** Machine learning model to diagnose PCG.

		AUC	Sensitivity	Specificity	PPV	NPV	Accuracy	F1
RF	all	0.72	0.57	0.64	0.64	0.59	0.6	0.59
	TAS	0.7	0.62	0.59	0.62	0.62	0.6	0.61
	SOD	0.56	0.57	0.5	0.55	0.53	0.54	0.56
	MDA	0.49	0.42	0.43	0.46	0.36	0.43	0.43
LR	tall	0.66	0.57	0.61	0.65	0.59	0.59	0.58
	TAS	0.6	0.7	0.38	0.55	0.53	0.54	0.61
	SOD	0.56	0.75	0.32	0.54	0.56	0.54	0.62
	MDA	0.66	0.62	0.56	0.62	0.57	0.59	0.61
KNN	all	0.54	0.47	0.63	0.57	0.54	0.55	0.52
	TAS	0.74	0.78	0.61	0.68	0.73	0.7	0.72
	SOD	0.56	0.55	0.58	0.57	0.56	0.57	0.56
	MDA	0.45	0.42	0.4	0.43	0.39	0.41	0.42
SVM	all	0.44	0.93	0.06	0.51	nan	0.5	0.65
	TAS	0.47	0.55	0.63	0.62	0.57	0.59	0.58
	SOD	0.44	0.82	0.32	0.56	0.68	0.58	0.66
	MDA	0.4	0.78	0.35	0.56	nan	0.57	0.64
Adaboost	all	0.68	0.5	0.64	0.65	0.51	0.57	0.55
	TAS	0.66	0.65	0.53	0.6	0.62	0.59	0.61
	SOD	0.51	0.57	0.44	0.52	0.51	0.51	0.54
	MDA	0.5	0.4	0.4	0.42	0.37	0.4	0.41
XGBoost	all	0.59	0.53	0.59	0.59	0.53	0.55	0.54
	TAS	0.74	0.7	0.69	0.7	0.7	0.69	0.7
	SOD	0.59	0.6	0.55	0.58	0.58	0.58	0.58
	MDA	0.46	0.53	0.5	0.49	0.53	0.51	0.50

Abbreviations: AB:adaptive boosting; MLP:multilayer perceptron; DT:decision tree; LGB: light gradient boost; KNN:k-nearest neighbors; GB:gradient boosting; RF: random forest; SVM:support vector machine; XGB:extreme gradient boosting; and GNB: Gaussian naive Bayes.

## 4. Discussion

In our case-control study, we identified a trio of biomarkers that decreases TAS, elevates H_2_O_2_ and increases MDA and that can independently predict the risk of PCG. Logistic regression revealed that serum TAS is a significant risk factor of PCG, a finding corroborated by the heightened levels of the oxidative stress indicators H_2_O_2_ and MDA. The research underscores the essential role of an oxidative stress imbalance in the causation and escalation of PCG. Furthermore, employing a five-fold cross-validation machine learning approach, we confirmed the predictive potential of TAS, alongside that of SOD and MDA, as a biomarker for congenital glaucoma. As such, our results pave the way for the early intervention strategies integrating neonatal biomarker screening and risk stratification, the intake of antioxidant-rich foods or supplements and the development of targeted antioxidant therapies (e.g., SOD mimetics),etc., while emphasizing the urgency of translational studies to validate therapeutic efficacy through in vivo models and clinical trials addressing redox homeostasis restoration in PCG patients.

Recent studies have underscored the link between oxidative stress and the progression of glaucoma. For instance, Yun Zhao et al. [14] highlighted the role of Cyp1b1 in the anterior segment’s development, emphasising its expression and function as crucial to the disease’s pathology. In response, the Cyp1b1-deficient (Cyp1b1 − /−) mouse model has emerged as a valuable tool for studying PCG induced by oxidative stress, as it demonstrates that Cyp1b1 is a significant regulator of oxidative balance, playing a key role in the development and maintenance of the trabecular meshwork structure and its function.

Recent studies have consistently found that biomarkers of oxidative stress are present in the aqueous humour [10,15] and peripheral blood [11,16–19] of glaucoma patients. Among them, the results suggested that serum MDA level was significantly increased and TAS significantly decreased in POAG, which were consistent with our results. In addition, Fran et al. reported the presence of oxidative stress biomarkers in the eye tissues of human glaucoma and experimental models [20,21], and Engin et al. [22] reported that serum levels of TAS, SOD and MDA are significantly elevated in these patients, pointing to a disrupted oxidative balance in the periphery. This finding is further supported by Nucci et al. [14], who observed elevated MDA and reduced TAS in aqueous humour. On the other hand, our previous study suggested that decreased levels of TAS and SOD as well as increased levels of MDA at baseline were associated with VF progression in patients with PACG, which is consistent with our results in this paper [23]. Meanwhile, McElnea et al. [20] extended these insights by demonstrating heightened ROS, particularly MDA, within the lamina cribrosa of glaucomatous eyes, contrasting normal eyes.

The relationship between the changes in the oxidative stress response and PCG must be fully studied. This study marks the first investigation into the correlation between oxidative stress biomarkers and PCG severity, revealing associations of low serum TAS and elevated H_2_O_2_ and MDA levels with disease severity. While there is limited literature on the prognostic value of these markers in PCG, an increasing body of evidence suggests that TAS, SOD and MDA may serve as potential biomarkers for disease progression and survival across various conditions [24–28]. For instance, Chen et al. [29] demonstrated a correlation between elevated SOD activity and reduced all-cause mortality in elderly women. Furthermore, a meta-analysis of five studies revealed a significant inverse association between dietary TAS levels and mortality from all causes, cancer and cardiovascular disease, highlighting the broad relevance of oxidative stress markers in health outcomes [26].

While the precise role of oxidative stress in glaucoma progression remains to be fully understood, it is known to arise from an imbalance between ROS and RNS production and the cell’s antioxidant defences [6,9]. Both environmental and endogenous stressors contribute to ROS and RNS overproduction, which, under normal conditions, are involved in vital cellular signalling processes [30,31]. However, when oxidant levels exceed homeostatic thresholds, they can cause direct cellular damage and activate pathways that lead to morphological and functional impairments, potentially influencing the pathogenesis of age-related diseases.

The body’s antioxidant defence primarily consists of enzymatic components, such as SOD, catalase and glutathione peroxidase, which work to neutralise ROS [32]. Our study found decreased TAS and increased H_2_O_2_ and MDA in patients with PCG, suggesting that the antioxidant system is overwhelmed by pro-oxidants, and this imbalance could lead to cellular damage, affecting the trabecular meshwork and retinal ganglion cells [33]. Importantly, our findings suggest that baseline TAS, H_2_O_2_ and MDA levels are significant predictors of PCG, implicating oxidative stress as a key factor in glaucoma onset.

As a pioneering study evaluating the relationship of serum levels of TAS, SOD and MDA with PCG, our research has several limitations. First, our findings are based on a single-centre case-control study involving Chinese participants, limiting the generalisability of our results to other ethnicities. In addition, the assessment of glaucoma severity relies not only on visual field and optic disc changes, but also on the extent of visual field loss, which could introduce confounding factors. Lastly, while we controlled for several potential confounders, such as age and sex, the influence of medication and smoking history on our results cannot be ruled out.

In conclusion, our study indicates that elevated serum H_2_O_2_ and MDA levels, in conjunction with reduced TAS, are linked to an increased risk of PCG. Furthermore, our analysis using machine learning algorithms suggests that TAS could serve as a potential biomarker for PCG. These findings implicate an oxidative stress imbalance as a factor contributing to the pathogenesis of PCG, highlighting its potential as a therapeutic target for glaucoma prevention and treatment.
